# Supplementary figures and images for: Infection of ratites with clade 2.3.4.4b HPAIV H5N1: potential implications for zoonotic risk
Source: Emerg Microbes Infect. 2026 Mar 13;15(1):2645853. doi: 10.1080/22221751.2026.2645853 (PMC13224690; doi:10.1080/22221751.2026.2645853)

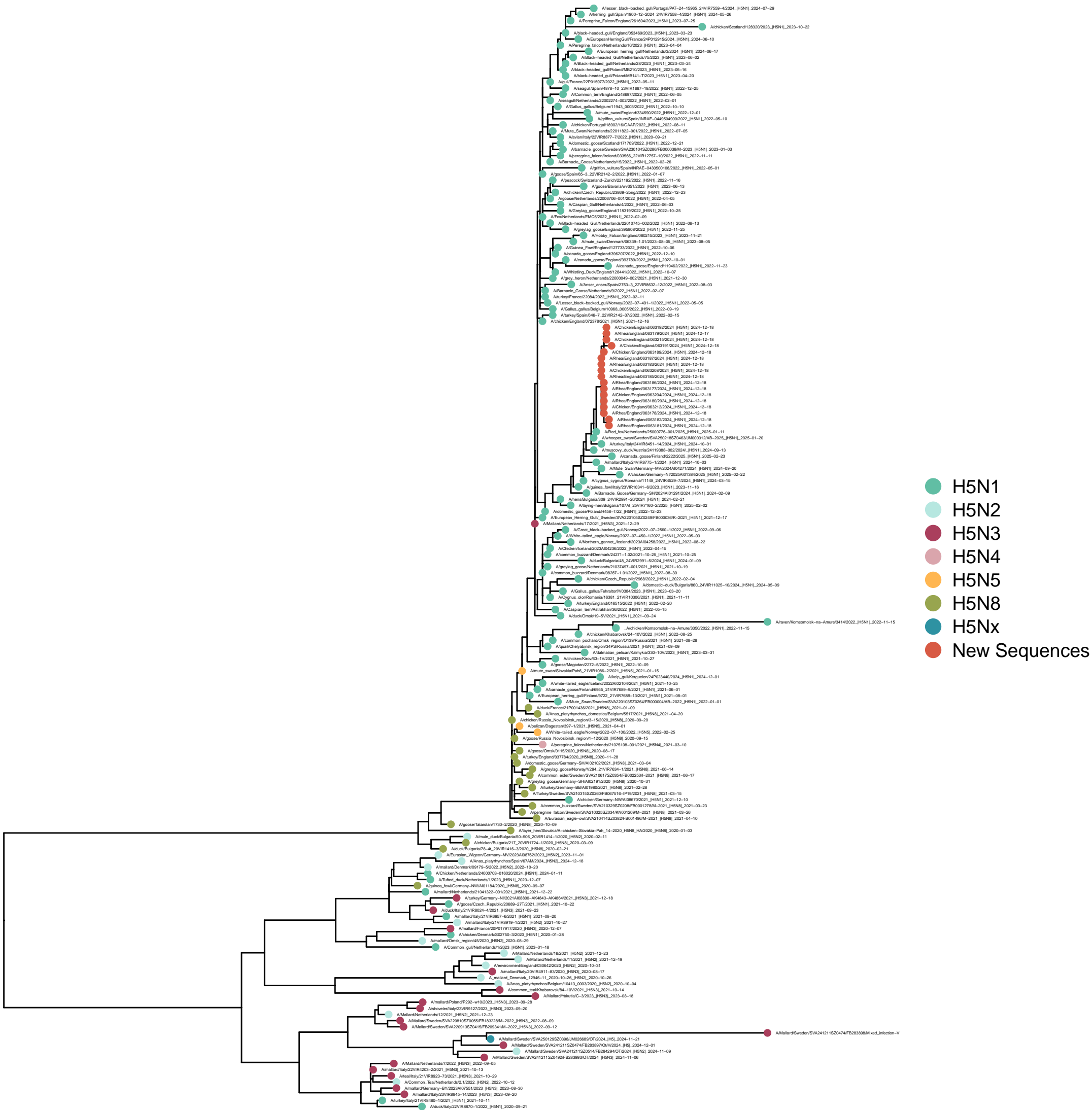

Supplement: Sup_Fig1A_HA_tree.pdf [file TEMI_A_2645853_SM5140.pdf]

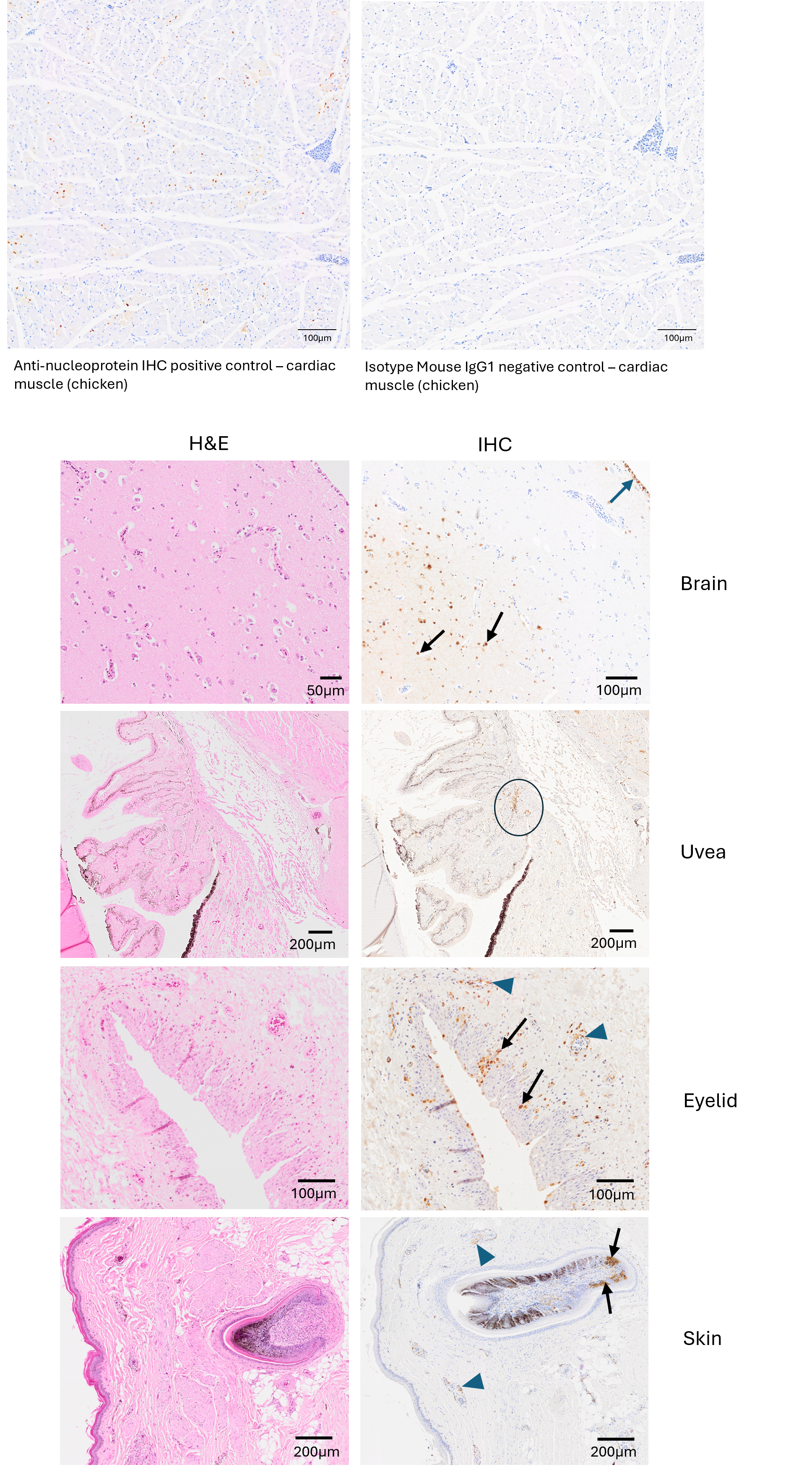

Supplement: Supplementary Figure 2.png [file TEMI_A_2645853_SM5138.png]

B

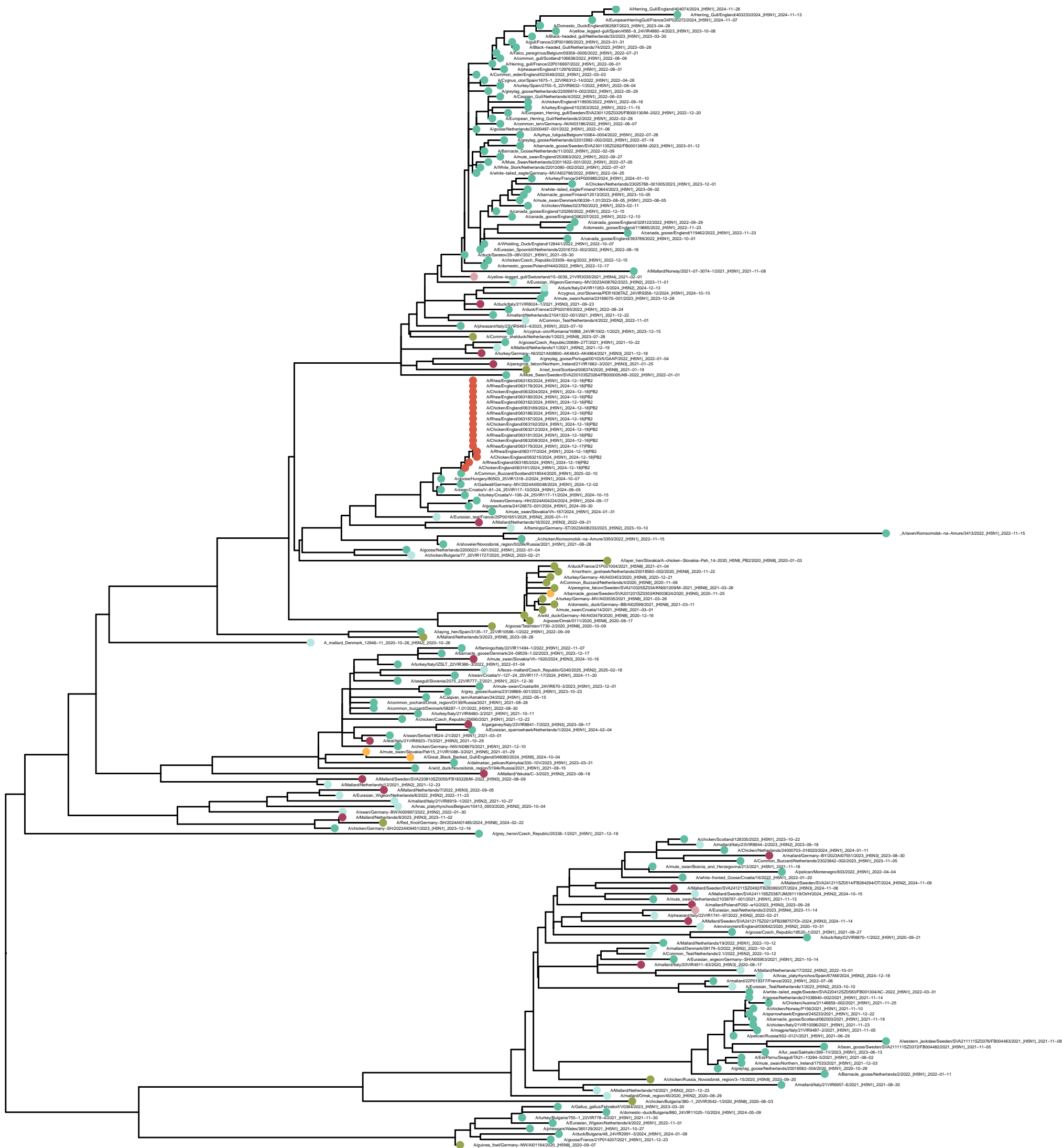

- H5N1
- H5N2
- H5N3
- H5N4
- H5N5
- H5N8
- New Sequences

Supplement: Sup_fig_1B_PB2_tree.pdf [file TEMI_A_2645853_SM5137.pdf]
