# Supplementary material for: Infection of ratites with clade 2.3.4.4b HPAIV H5N1: potential implications for zoonotic risk
Source: Emerg Microbes Infect. 2026 Mar 13;15(1):2645853. doi: 10.1080/22221751.2026.2645853 (PMC13224690; doi:10.1080/22221751.2026.2645853)
Supplement: Supplementary Materials.docx [file TEMI_A_2645853_SM5139.docx]

**Supplementary Table 1:** RT-PCR results and whole genome sequencing on samples collected from Rheas and Chickens housed on the infected premises

| **Species** | **Animal** | **Sample Type** | **RT-PCR result** | | |  |  |  |  |  |
| --- | --- | --- | --- | --- | --- | --- | --- | --- | --- | --- |
|  |  |  | **(M-gene)** | **H5HP** | **N1** | **EURL Genotype** | **PB2-627** | **PB2-701** | **Strain** | **Gisaid EPI ID** |
| Rhea | 1 | OP swab | **Positive** | **Positive** | **Positive** | DI.2 | K | D | A/Rhea/England/063177/2024_\|H5N1\|_2024-12-18 | EPI_ISL_20156266 |
|  |  | C swab | **Positive** | **Positive** | **Positive** | DI.2 | K | D | A/Rhea/England/063178/2024_\|H5N1\|_2024-12-18 | EPI_ISL_20354342 |
|  |  | Brain | **Positive** | **Positive** | **Positive** | DI.2 | K | D | A/Rhea/England/063179/2024_\|H5N1\|_2024-12-18 | EPI_ISL_20156402 |
|  | 2 | OP swab | **Positive** | **Positive** | **Positive** | DI.2 | K | D | A/Rhea/England/063180/2024_\|H5N1\|_2024-12-18 | EPI_ISL_20156403 |
|  |  | C swab | **Positive** | **Positive** | **Positive** | DI.2 | K | D | A/Rhea/England/063181/2024_\|H5N1\|_2024-12-18 | EPI_ISL_20156404 |
|  |  | Brain | **Positive** | **Positive** | **Positive** | DI.2 | K | D | A/Rhea/England/063182/2024_\|H5N1\|_2024-12-18 | EPI_ISL_19659601 |
|  | 3 | OP swab | **Positive** | **Positive** | **Positive** | DI.2 | K | D | A/Rhea/England/063183/2024_\|H5N1\|_2024-12-18 | EPI_ISL_19897983 |
|  |  | C swab | **Positive** | **Positive** | **Positive** | DNS^-^ | DNS |  |  |  |
|  |  | Brain | **Positive** | **Positive** | **Positive** | DI.2 | E | D | A/Rhea/England/063185/2024_\|H5N1\|_2024-12-18 | EPI_ISL_20156406 |
|  | 4 | OP swab | **Positive** | **Positive** | **Positive** | DI.2 | K | D | A/Rhea/England/063186/2024_\|H5N1\|_2024-12-18 | EPI_ISL_20156407 |
|  |  | C swab | **Positive** | **Positive** | **Positive** | DI.2 | K | D | A/Rhea/England/063187/2024_\|H5N1\|_2024-12-18 | EPI_ISL_19659622 |
| Chicken | 5 | OP swab | Negative | Negative | Negative |  |  |  |  |  |
|  |  | C swab | **Positive** | **Positive** | **Positive** | DI.2 | K | D | A/Chicken/England/063189/2024_\|H5N1\|_2024-12-18 | EPI_ISL_19897983 |
|  | 6 | OP swab | Negative | Negative | Negative |  |  |  |  |  |
|  |  | C swab | **Positive** | **Positive** | **Positive** | DI.2 | E | D | A/Chicken/England/063191/2024_\|H5N1\|_2024-12-18 | EPI_ISL_19897607 |
|  | 7 | OP swab | Negative | **Positive** | **Positive** | DI.2 | K | D | A/Chicken/England/063192/2024_\|H5N1\|_2024-12-18 | EPI_ISL_20156408 |
|  |  | C swab | Negative | Negative | Negative |  |  |  |  |  |
|  | 8 | OP swab | Negative | Negative | Negative |  |  |  |  |  |
|  |  | C swab | Negative | Negative | Negative |  |  |  |  |  |
|  | 9 | OP swab | Negative | Negative | Negative |  |  |  |  |  |
|  |  | C swab | Negative | Negative | Negative |  |  |  |  |  |
|  | 10 | OP swab | Negative | Negative | Negative |  |  |  |  |  |
|  |  | C swab | Negative | Negative | Negative |  |  |  |  |  |
|  | 11 | OP swab | Negative | Negative | Negative |  |  |  |  |  |
|  |  | C swab | Negative | Negative | Negative |  |  |  |  |  |
|  | 12 | OP swab | Negative | Negative | Negative |  |  |  |  |  |
|  |  | C swab | Negative | Negative | Negative |  |  |  |  |  |
|  | 13 | OP swab | Negative | **Positive** | Negative | DI.2 | K | D | A/Chicken/England/063204/2024_\|H5N1\|_2024-12-18 | EPI_ISL_20156267 |
|  |  | C swab | Negative | Negative | Negative |  |  |  |  |  |
|  | 14 | OP swab | Negative | Negative | Negative |  |  |  |  |  |
|  |  | C swab | Negative | Negative | Negative |  |  |  |  |  |
|  | 15 | OP swab | Negative | **Positive** | Negative | DI.2 | K | D | A/Chicken/England/063208/2024_\|H5N1\|_2024-12-18 | EPI_ISL_20156268 |
|  |  | C swab | Negative | Negative | Negative |  |  |  |  |  |
|  | 16 | OP swab | Negative | Negative | Negative |  |  |  |  |  |
|  |  | C swab | Negative | Negative | Negative |  |  |  |  |  |
|  | 17 | OP swab | **Positive** | **Positive** | **Positive** | DI.2 | K | D | A/Chicken/England/063212/2024_\|H5N1\|_2024-12-18 | EPI_ISL_20156269 |
|  |  | C swab | Negative | Negative | Negative |  |  |  |  |  |
|  | 18 | OP swab | Negative | Negative | Negative |  |  |  |  |  |
|  |  | C swab | Negative | **Positive** | Negative | DI.2 | K | N | A/Chicken/England/063215/2024_\|H5N1\|_2024-12-18 | EPI_ISL_20156409 |

*OP= oropharyngeal; C=cloacal; ^-^DNS=did not produce a sequence

**Supplementary Table 2:** Amino acid changes in the sequences generated in this study when compared to UK DI.2 sequences sampled one month preceding or subsequent to the outbreak in the Rheas. Gene segments not listed contained no amino acid changes.

| **Strain** | **Gisaid EPI ID** | **HA** | **PB1-F2** | **M2** | **NP** | **PA** | |
| --- | --- | --- | --- | --- | --- | --- | --- |
| **Amino Acid Position** |  | **15** | **50** | **10** | **52** | **428** | **61** |
| A/Rhea/England/063177/2024_\|H5N1\|_2024-12-18 | EPI_ISL_20156266 | R | G | H | H | V | K |
| A/Rhea/England/063178/2024_\|H5N1\|_2024-12-18 |  | R | G | H | H | V | K |
| A/Rhea/England/063179/2024_\|H5N1\|_2024-12-18 | EPI_ISL_20156402 | R | G | H | H | V | K |
| A/Rhea/England/063180/2024_\|H5N1\|_2024-12-18 | EPI_ISL_20156403 | R | G | H | H | V | K |
| A/Rhea/England/063181/2024_\|H5N1\|_2024-12-18 | EPI_ISL_20156404 | R | G | H | H | V | K |
| A/Rhea/England/063182/2024_\|H5N1\|_2024-12-18 | EPI_ISL_19659601 | R | G | H | H | V | T |
| A/Rhea/England/063183/2024_\|H5N1\|_2024-12-18 | EPI_ISL_19897983 | R | G | H | H | V | T |
| A/Rhea/England/063185/2024_\|H5N1\|_2024-12-18 | EPI_ISL_20156406 | R | G | H | H | V | K |
| A/Rhea/England/063186/2024_\|H5N1\|_2024-12-18 | EPI_ISL_20156407 | R | G | H | H | V | K |
| A/Rhea/England/063187/2024_\|H5N1\|_2024-12-18 | EPI_ISL_19659622 | R | G | H | H | V | K |
| A/Chicken/England/063189/2024_\|H5N1\|_2024-12-18 | EPI_ISL_19897983 | R | G | H | H | V | T |
| A/Chicken/England/063191/2024_\|H5N1\|_2024-12-18 | EPI_ISL_19897607 | K | G | H | H | V | - |
| A/Chicken/England/063192/2024_\|H5N1\|_2024-12-18 | EPI_ISL_20156408 | R | G | H | H | V | T |
| A/Chicken/England/063204/2024_\|H5N1\|_2024-12-18 | EPI_ISL_20156267 | R | G | H | H | V | - |
| A/Chicken/England/063208/2024_\|H5N1\|_2024-12-18 | EPI_ISL_20156268 | R | G | H | H | V | K |
| A/Chicken/England/063212/2024_\|H5N1\|_2024-12-18 | EPI_ISL_20156269 | R | G | H | H | V | T |
| A/Chicken/England/063215/2024_\|H5N1\|_2024-12-18 | EPI_ISL_20156409 | R | G | H | H | V | K |
| UK DI.2* |  | K | D | P | Y | I | T |

*UK DI.2 sequences from wild birds and poultry sampled within a month of the outbreak reported in this manuscript.

**Supplementary Figure 1:** Maximum-likelihood HA (**A**) and PB2 (**B**) phylogeny of European H5 influenza sequences from GISAID, subset to 0.5% sequence divergence, used to contextualise how sequences generated in this study compare to previously released European data. Tip labels are coloured by subtype.

**Supplementary Figure 2**: Representative histopathologic and immunohistochemical findings in brain, eye (uvea), eyelid and skin of a rhea. Positive labelling in neurons (black arrows) and ependymal cells (blue arrows, brain), epithelial cells of the ciliary body (black circle, eye) and conjunctiva (black arrows, eyelid), and epithelium of the feather (black arrows, skin). Positive labelling of vascular endothelium is also evident (blue arrowheads).
